# Optimization of Split Feeding Strategy for Laying Hens Through a Response Surface Model

**DOI:** 10.3390/ani15050750

**Published:** 2025-03-05

**Authors:** Nasima Akter, Thi Hiep Dao, Tamsyn M. Crowley, Amy F. Moss

**Affiliations:** 1 School of Environmental and Rural Science, Faculty of Science, Agriculture, Business and Law, University of New England, Armidale, NSW 2351, Australia or shumi.cvasu13@cvasu.ac.bd (N.A.); tdao2@une.edu.au (T.H.D.); 2 Department of Dairy and Poultry Science, Faculty of Veterinary Medicine, Chattogram Veterinary and Animal Sciences University, Chattogram 4225, Bangladesh; 3 Institute for Mental and Physical Health and Clinical Translation (IMPACT), School of Medicine, Deakin University, Geelong, VIC 3220, Australia; tamsyn.crowley@une.edu.au

**Keywords:** AM/PM feeding, split feeding, laying hens, feed efficiency, egg quality

## Abstract

Laying hens’ metabolism goes through a cyclic process to produce eggs, which requires higher dietary protein and energy in the morning (AM) and higher calcium (Ca) in the afternoon/evening (PM) than the rest of the day. Therefore, poultry scientists are trying to adopt a new feeding strategy called AM/PM or split feeding to precisely meet hen’s requirements more effectively than conventional methods. A 10-week cage layer trial was carried out via a Box–Behnken response surface design to identify the optimal amount of protein, energy, and calcium of the AM/PM diets. There were 13 test treatments with three levels of crude protein (19.6%/18.4%, 20.3%/17.7%, 21%/17%), calcium (3.3%/4.9%, 2.5%/5.7%, 1.6%/6.6%), and apparent metabolizable energy (AME) (12 MJ/kg/11.2 MJ/kg, 12.4 MJ/kg/10.8 MJ/kg, 12.8 MJ/kg/10.4 MJ/kg) for AM/PM diets respectively and a control treatment with industry baseline (CP-19%, Ca-4.1% and ME 11.6 MJ/kg). These are the calculated values of nutrients on a dry matter basis. A total of 364 hens were randomly distributed into 2 dietary treatments where each treatment had 13 replicates (2 hens per replicate cage, 26 hens per treatment). AM and PM diets were swapped out at approximately 8 am and 4 pm each day. Egg production and hen performance were measured daily and weekly, respectively, with egg quality, serum Ca, and nutrient digestibility measured at week 10. AM:PM intake and feed cost were calculated for each treatment. The optimal FCR, feed cost, and AM:PM intake were used to determine Ca, CP, and AME levels. The result showed that 6 out of 13 of our test treatments gave improved FCR compared to the control treatment (*p* = 0.017). Dietary treatments did not affect overall hen weight and serum Ca and egg quality at week 10, except for the lower yolk color score in the control treatment (*p* = 0.002). Hens in the experimental treatment, with calcium levels of 1.6% and 6.6%, crude protein (CP) levels of 19.6% and 18.4%, and AME content of 12.4 MJ/kg and 10.8 MJ/kg in the AM and PM diet, respectively, showed the highest apparent protein digestibility (56.6%) compared to the control group (*p* < 0.05). Similarly, hens receiving a treatment containing calcium at 3.3% and 4.9%, CP at 21% and 17%, and AME at 12.4 MJ/kg and 10.8 MJ/kg in the AM and PM diet, respectively, achieved the highest calcium digestibility (62.13%), while the control treatment yielded the lowest calcium digestibility (*p* < 0.05). After analyzing the data using the Box–Behnken response surface methodology, we found that (21/17)% CP, (3.3/4.9)% Ca, and (12/11.12) MJ/kg energy in the AM/PM diet gave the optimum performance in terms of lower feed cost and better feed efficiency. The data of AM:PM intake demonstrate that selective feeding occurs in between treatments (*p* < 0.001) and the degree of selection depends on the difference between the level of nutrients in AM and PM diet. This study revealed that when optimized, AM/PM feeding improves feed efficiency and egg quality of laying hens.

## Simple Summary

Hens require varying nutrient levels throughout the day due to their cyclic reproductive physiology. High levels of dietary protein and energy are essential in the morning to support yolk and albumen formation, while high calcium intake is crucial in the afternoon and evening to facilitate eggshell and membrane development. Feeding a single diet throughout the day can result in nutrient imbalances, with excess calcium in the morning and surplus protein, amino acids, and energy in the afternoon. To address this, the split feeding strategy, also known as morning and afternoon (AM/PM) feeding, involves providing a high-protein, high-energy, low-calcium diet in the morning, followed by a low-protein, low-energy, high-calcium diet in the afternoon or evening. This tailored feeding strategy optimizes nutrient utilization and enhances production efficiency. The study findings revealed that hens on AM/PM diets demonstrated improved feed efficiency, enhanced yolk coloration, and reduced feed costs, supporting the effectiveness of this approach in layer production systems.

## 1. Introduction

The idea of precision agriculture has swiftly expanded due to technological advancements and has been adopted in different agricultural systems, leading to reduced expenses, enhanced yields, and support for more sustainable methods [1]. Before this, technology was focused mainly on animal farming with greater investments and higher feeding expenses because of the significant initial costs. However, with increasing economic difficulties such as volatile egg prices [2] and the fact that feed accounts for over 65% of the costs associated with live poultry production [3], nutritional strategies to more precisely meet poultry nutrient requirements are becoming essential for economic sustainability.

AM/PM feeding, also known as split feeding, for layer hens is one such strategy that aims to make a relatively simple adjustment in the way hens are fed to achieve precision nutrition. Conventional laying hen practices offer a single complete diet to hens throughout the whole day. However, egg formation is a cyclic process that leads to different nutrient requirements in the morning compared to the afternoon/evening [4,5]. For example, in the morning, hens lay eggs from the previous day, ovulate the next day’s yolk, and then lay down the egg white or albumen around that yolk [6,7,8]. These actions require a higher level of protein [6,8,9]. In the afternoon, hens lay the egg shell, which requires a higher level of calcium [10,11]. Therefore, the common practice of offering hens only a single complete feed with the average protein and calcium throughout the whole day may not provide enough protein in the morning or enough calcium in the evening [12,13]. Also, an unnecessarily higher calcium level in the morning may worsen the digestibility of other nutrients [14,15], so minimizing it in the morning may also promote protein digestibility and reduce the amount of wasted nutrients.

So, to improve profitability and sustainability, egg producers are considering AM/PM feeding, also known as split feeding. The principle is that hens are fed an AM and a PM ration, each tailored to the nutrition the birds need at that time of the day, which involves feeding a high-protein diet in the morning and a high-calcium diet in the evening [16,17,18,19,20]. Since the retention time of feed in the digestive tract of poultry is notably brief, averaging approximately 5 to 6 h [21], providing separate AM and PM diets may help chickens acquire the specific nutrients needed at different times of the day. This AM/PM feeding strategy would provide production and economic benefits like improved egg quality and skeletal health of layers. Moreover, as the AM ration requires less limestone, there is more room for fiber, which will make hens satiated (feel fuller) in the middle of the day and, thus, help to prevent cannibalism by stopping them from pecking each other [22,23,24]. So, by accurately meeting nutrient demands, we may also see a reduced incidence of cannibalism and, therefore, better welfare for laying hens.

This AM/PM approach does not need a large investment in technology to implement; instead, it leverages the natural biological rhythms of the hen. For example, layer facilities (cage or free range) are already equipped with feeder lines within the sheds and may have one or two silos. Investment for a second silo leading into the feeder line may be required if a farm only has one. From the two silos leading into the feeder lines, the hens may be offered the AM and PM diets at their respective times of day. Thus, AM/PM feeding for layer hens is a rapidly implementable strategy to introduce precision nutrition to the Australian layer industry for improved efficiency of production, improved egg quality, reduced environmental impact, and positive welfare benefits.

Probably the first account of AM/PM feeding was that of Penz and Jensen [8], who identified that hens require more protein in their diet following oviposition. Following this, further studies explored manipulating both dietary protein and calcium (Ca) levels [25,26,27,28,29,30], as reviewed by Molnár et al. [4]. Within many of these reports, it was concluded that reducing dietary Ca content in the morning improved feed conversion [29,30] or the reduced dietary Ca level had no effect on egg shell quality [25,26,27,28]. Additionally, Mozos et al. [30] demonstrated that energy and protein may be reduced in the afternoon feed, which should present substantial cost savings for producers. By providing the nutrients when they are required, it is hypothesized that it may help to reduce cannibalism and feather pecking, which can be affected by insufficient protein [31]. Furthermore, keel bone fractures are not only a welfare issue but also reduce egg production [32]. Thus, given the above, it is sensible to hypothesize that, by improving calcium uptake when it is required, AM/PM feeding may also improve production and welfare through improved bone strength, resulting in fewer keel bone fractures. Therefore, there are positive indications in the literature that this strategy would be of benefit to the poultry industry. However, the optimum levels three major nutrients i.e., Ca, protein and energy for AM/PM feeding regime in laying hens are yet to be determined. So, this study was conducted to identify the optimal amount of protein, energy and calcium of the AM/PM diets for laying hens and to determine if selective feeding occurs across different levels of these nutrients. A Box–Behnken response surface design was utilized in this study to optimize nutrient levels, as it is widely used in poultry nutrition research. This design efficiently assesses multiple nutrient levels while minimizing the number of treatments and animals required, making it a practical and effective approach [33].

## 2. Materials and Methods

The study took place at the cage layer facility of Laureldale Research Station, University of New England, Armidale, NSW, Australia, using Hy-Line Brown laying hens. The experimental design and procedures received approval from the University of New England Animal Ethics Committee (approval number: ARA21-105) and adhered to the Australian Code of Practice for the Care and Use of Animals for Scientific Purposes [34].

### 2.1. Animal Husbandry and Birds

A total of 364 Hy-Line Brown pullets were sourced at 15 weeks of age (WOA) from a commercial layer farm in Tamworth, NSW, Australia. The birds were randomly placed into 182 cages, each housing two birds, with dimensions of 30 cm width × 50 cm depth × 45 cm height, inside a curtain-sided experimental shed. Upon arrival, the pullets were fed a standard laying hen diet (Barastoc—Premium Top Layer Mash, formulated to meet breed-specific nutrient requirements, containing 16.5% crude protein, 2.5% crude fat, 6% crude fiber, 0.3% salt, 8.0 mg/kg copper, 0.3 mg/kg selenium, and 3.6% calcium; Ridley Corp. Ltd., Melbourne, VIC, Australia) and allowed to adapt to their new environment until 21 WOA. The hens were weighed and assigned to experimental treatments from weeks 22 to 31. Feed and water were available ad libitum throughout the study, with each cage equipped with one feed trough and one nipple drinker. Lighting was provided using specialized poultry white LED bulbs (IP65 Dimmable LED Bulb, B-E27:10W, 5K; Eco Industrial Supplies, Zhenjiang, China) and maintained on a 16-h light and 8-h dark cycle, with lights turning on at 5 a.m. and off at 9 p.m.

**Figure 1:**
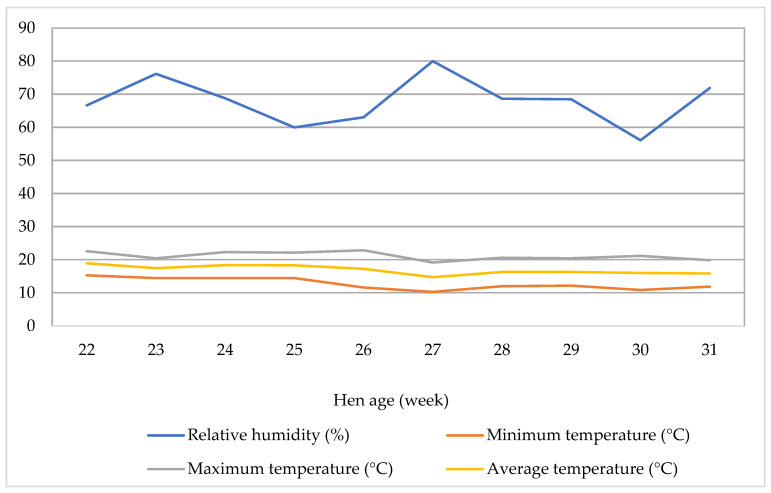
Temperature and relative humidity of the hen house during 10 weeks of study.

The temperature and relative humidity inside the shed were monitored daily, both in the morning and evening, at bird height using a thermometer/hygrometer (Temp Alert, FCC RoHS, 2011/65/EU, FCC: R17HE910, S4GEM35XB, Boston, MA, USA). Figure 1 displays the average ambient temperature (°C) and relative humidity (%) inside the shed from week 1 to 10. Over the study period, the mean air temperature was 17 °C, with a range of 10 °C to 23 °C, and the average relative humidity was 68%, varying from 56% to 80%. The maximum temperature ranged from 19 °C to 23 °C, with an average of 21 °C, while the minimum temperature ranged from 10 °C to 15 °C, averaging 13 °C.

### 2.2. Dietary Treatments and Study Design

This 10-week study employed a Box–Behnken response surface design to determine the optimal levels of crude protein, apparent metabolizable energy, and calcium in AM/PM diets for laying hens aged 22 to 31 weeks. The Box–Behnken Design (BBD), a commonly applied method in poultry nutrition research, is highly effective for evaluating multiple nutrient levels while reducing the number of treatments and animals required [33]. The authors chose this design due to its several advantages, including the ability to study multiple nutrients simultaneously with fewer treatments compared to a full factorial design, thereby reducing the number of birds used and aligning with key principles of animal ethics. This model has been successfully implemented in various poultry nutrition studies, and its efficacy has been underscored in a published review highlighting its utility in this field [33]. For instance, the current study utilized three levels of three factors—crude protein, calcium, and apparent metabolizable energy—arranged in a Box–Behnken Design as detailed in Table 1, Table 2 and Table 3. This approach resulted in 13 treatments instead of the 27 treatments that would have been required in a full factorial design [35,36]. In the BBD, specific design points with extremely high or low factor levels are excluded from a full factorial design, as they are assumed to be unnecessary for optimization. This is based on the expectation that the minimum or maximum levels of the factors will produce the minimum or maximum desired response, and the optimal response is unlikely to be found in these extreme factor levels. The BBD utilizes a spread of points to optimize a given parameter via a regression equation, but it does so while removing points which are not required to still generate a response surface. A key advantage of BBD is its efficiency, as it requires fewer treatment combinations. However, this approach may not be suitable if the objective is to investigate extreme responses to factors [33]. In addition to the 13 experimental treatments, a control treatment (treatment 14) was included, featuring nutrient levels typically used for laying hen ration in the industry (Table 3). The study utilized 13 replicates per treatment, with two hens housed per replicate cage, resulting in 26 hens per treatment and 364 hens in total. Housing two hens per cage helped reduce variability between cages and enhanced the statistical power of the study.

The composition of the experimental treatments is detailed in Table 4 and Table 5. Here, treatment 14 represents the control diet, which was formulated based on the standard industry baseline commonly used in commercial laying hen production. It is important to include this as a control, as industries often require comparative results to evaluate how the new strategy performs relative to the existing one. All diets were prepared as mash at the UNE Centre for Animal Research and Teaching feed mill facility. Nutritional parameters such as dry matter (DM), apparent metabolizable energy (AME), crude protein (CP), digestible lysine, calcium (Ca), phosphorus (P), and sodium (Na) in the primary feed ingredients were analyzed using near-infrared reflectance spectroscopy (Foss NIR 6500, Hillerød, Denmark), standardized with Evonik AMINONIR Advanced calibration, to formulate the experimental diets. The calculated nutritional values of dietary treatments are shown in Table 6. Furthermore, the actual nutrient content of the prepared diets, including DM, gross energy (GE), CP, Ca, P, and Na, was assessed using standard analytical methods [37] and is reported in Table 7. For feeding, the control diet was provided to hens continuously throughout the day, while the AM and PM diets were alternated daily at approximately 8 a.m. and 4 p.m., respectively.

### 2.3. Data and Sample Collection

To ensure uniformity, the birds were weighed before being assigned to dietary treatments, confirming no significant differences in initial weights among treatments (*p* > 0.05). Hen body weights were recorded at weeks 5 and 10 of the trial to calculate weight gain over time. Daily records were kept for egg number and weight, while internal and external egg quality were evaluated at the end of the trial (week 10). Feed intake was monitored weekly, with AM and PM feed consumption recorded separately to calculate the AM/PM intake ratio. This approach helped to assess whether hens could differentiate and select between the two diets. It was hypothesized that the most distinct AM and PM diet formulations would make it easier for hens to distinguish and choose between the AM and PM components. Total feed intake was calculated by adding up the weekly intakes of AM and PM diets of the AM/PM treatment cages (containing two hens per cage). Total intakes of CP, energy, and Ca were calculated using data from the analyzed nutrient value of the AM/PM diets. The nutrient levels yielding the best feed conversion ratio (FCR) and lowest feed costs were identified to establish the optimal combination of calcium (Ca), crude protein (CP), and apparent metabolizable energy (AME). These optimal levels were then applied in a subsequent 20-week free-range laying hen trial. Performance indices such as hen-day egg production (HDEP), egg mass, FCR, AM/PM intake ratio, and feed cost were calculated using the following equations:

$$HDEP\left(\%\right)=\frac{Total\;number\;of\;eggs}{Total\;number\;of\;hens\times 7\left(days\right)}\times 100$$

Egg mass (g/day)=HDEP (%)×Average egg weight (g)

$$FCR=\frac{kg\;of\;feed\;consumed}{kg\;of\;egg\;mass}$$

$$AM:PM\;intake=\frac{kg\;of\;AM\;feed\;intake}{kg\;of\;PM\;feed\;intake}$$

$$Feed\;cost\;per\;kg\;egg\;mass=\frac{Total\;feed\;intake\;\left(kg\right)\times Feed\;cost\;(AUD)}{Total\;egg\;mass\;\left(kg\right)}$$

Total excreta samples were collected over the first three consecutive days of week 10 to evaluate the apparent digestibility of nutrients. The feed consumption of individual cages (containing two hens per cage) was recorded during the 3-day excreta collection period. Blood samples were also taken at the same time (week 10) to measure serum Ca levels.

### 2.4. Egg Quality Assessment

To evaluate the internal and external quality of eggs, 182 eggs (one egg per cage, 13 eggs per treatment) were collected in the morning at week 10 and transported to the laboratory. Except for eggshell weight and thickness (which required drying before measurement), all quality parameters were assessed within four hours of collection. Egg length (mm) and width (mm) were measured using a digital Vernier caliper (Kincrome^®^, 0–150 mm scale, Scoresby, VIC, Australia) to calculate the egg shape index (SI = width/length × 100). Eggshell reflectivity was determined using a shell reflectivity meter (Technical Services and Supplies, Dunnington, York, UK), while eggshell breaking strength and internal egg quality parameters were analyzed with a digital egg tester (DET6500^®^, Nabel Co., Ltd., Kyoto, Japan). Yolks were separated from albumen using Whatman filter papers (CAT No. 1541–090, Whatman^®^, Buckinghamshire HP7 9NA, Amersham, UK) and weighed. Albumen weight was calculated by subtracting the yolk and eggshell weights from the total egg weight. Eggshells were cleaned, air-dried for at least 72 h, and weighed using a precision analytical balance (Adventurer TM, Model AX423, Ohaus^®^, Newark, NJ, USA). Thickness, including the outer shell membrane, was measured using a custom-built gauge (Mitutoyo Dial Comparator Gauge, Model 2109-10, Kawasaki, Japan).

### 2.5. Serum Ca Analysis

A subset of hens (3 cages or 6 hens per treatment, totaling 84 hens) was selected for blood sample collection based on body weights closest to the average body weight for each treatment. Blood was drawn from the wing veins and transferred into silica-coated vacutainers (Becton, Dickinson UK Limited, Plymouth, UK) containing serum separator polymer gel for serum calcium (Ca) analysis. The samples were immediately transported to the laboratory in a cool box. Upon arrival, the vacutainers were centrifuged at 3000× *g* at 4 °C for 10 min, and the serum supernatant was transferred into 2 mL micro-centrifuge tubes. The serum samples were then stored at −20 °C until further analysis. Serum calcium (Ca) levels were determined in duplicate using commercial kits (Reference number 981772, Thermo Fisher Scientific Inc., Waltham, MA, USA) with a Thermo Scientific Indiko Plus clinical chemistry analyzer (Thermo Fisher Scientific Inc., Waltham, MA, USA) according to the manufacturer’s guidelines. The measurements were subsequently read on a SpectraMax M2e plate reader (Molecular Devices, San Jose, CA, USA).

### 2.6. Apparent Digestibility of Nutrients

The gross energy (GE), crude protein (CP), and Ca and P digestibility of the dietary treatments were evaluated at week 10 of the trial. Hens (3 cages or 6 hens per treatment, 84 hens in total) having weights closer to average treatment weight were selected for excreta collection using the total collection method. Excreta, free from feathers, dirt, and feed, were collected from individual cages (separate trays used for each cage) each morning for three consecutive days (72 h) and stored in polypropylene zipper bags. The samples were subsequently transported to the laboratory, thoroughly mixed, and subsampled into 70 mL plastic containers for storage at 4 °C. Approximately 5 g of fresh excreta was weighed into pre-weighed crucibles and dried in a forced air oven (Qualtex, Solidstat Temperature Control Oven, Model No. OM24SE3, Morningside, QLD, Australia) at 105 °C for about 48 h to a constant weight for dry matter (DM) determination. The remaining subsamples were stored at −20 °C for further analysis. Frozen samples were later freeze-dried (Christ Alpha 1–4 LD Plus, Osterode am Harz, Germany) and then ground into fine particles using an ultra-centrifugal mill (Retsch ZM 200, Fisher Scientific, Hampton, NH, USA) with a 0.5 mm screen. Feed samples were also grounded similarly.

The protein concentration in feed and excreta was measured using the Dumas combustion method [38] with a nitrogen analyzer (LECO Corporation, St. Joseph, MI, USA), using EDTA as a calibration standard. GE concentration in feed and excreta was analyzed using a Parr Adiabatic Oxygen Bomb Calorimeter (Parr Instrument Co., Moline, IL, USA) with benzoic acid as the calibration standard. Feed samples were also oven-dried at 105 °C for approximately 24 h to a constant weight to determine dietary DM for calculating GE and CP digestibility on a DM basis. The mineral content in the excreta was measured using an inductively coupled plasma-optical emission spectrometry (ICP-OES) instrument (Agilent Technologies, Mulgrave, VIC, Australia) in accordance with the methodology outlined by Zanu et al. [39]. The apparent energy, protein, Ca, and P were calculated using equations provided by Kong and Adeola [40]:

Apparent protein digestibility (%) = (CP_retained/_CP_intake_) × 100

Apparent energy digestibility (%) = (GE_retained_/GE_intake_) × 100

Apparent mineral digestibility (%) = (Mineral_retained_/Mineral_intake_) × 100

CP_intake_ (g/day) = CP_feed_ (%) × FI (g/day/hen)

GE_intake_ (kcal/day) = GE_feed_ (kcal/g) × FI (g/day/hen)

Mineral_intake_ (g/day) = Mineral_feed_ × FI (g/day/hen)

CP_retained_ (g/day) = CP_intake_ − CP_excreta_ (%) × excreta volume (g/day/hen)

GE_retained_ (kcal/day) = GE_intake_ − GE_excreta_ (kcal/g) × excreta volume (g/day/hen)

Mineral_retained_ (g/day) = Mineral_intake_ − Mineral_excreta_ (%) × excreta volume (g/day/hen)

### 2.7. Data Analysis

After organizing the data in Microsoft Excel spreadsheets, data on feed cost, FCR, and AM/PM intake ratio were subjected to Box–Behnken response surface analysis because these are the important factors to choose an optimal AM/PM diet for industry (reduce feed cost). Additionally, we have included the ANOVA analysis for all the parameters as we have added an extra treatment to the study (control treatment 14); it is not part of the regression but instead serves as a commercial standard treatment to compare the overall performance back to. This is important because the industry is often enquiring as to how this strategy fares versus standard practice (to justify the cost of the equipment needed).

Using polynomial regressions in R version 3.3.3 and the RSM package, model predictions and response surface plots were produced from the experimental data. The reduced equations were recalculated for every response variable, and non-significant coefficients were eliminated during the model-generation process. When multiple significant models were found, the Akaike Information Criterion (AIC) was used. The selection of models was conducted according to the methods described by Liu et al. [36], which made sure that the models that were selected had the fewest number of parameters required to obtain the highest multiple R^2^ values, only significant parameters, and no significant lack of fit. Replica cage means were used to define the experimental units, and a probability level of less than 5% was used to evaluate statistical significance. A Pearson correlation was performed between FCEM and FCR.

ANOVA analyses were performed using IBM SPSS Statistics software (Version 28.0.1.0, IBM Corp., Armonk, NY, USA), with a significance level set at 0.05. Before conducting the statistical analysis, the data were tested for normality and homogeneity of variances across the dietary treatments. ANOVA was carried out using univariate General Linear Models (GLM), with treatment as a fixed effect, to determine mean differences between the treatments. Tukey’s post hoc test was used to identify pairwise differences when significant results were observed. The *p*-value of ≤0.05 was considered statistically significant, while values between 0.05 and 0.10 were interpreted as trends.

## 3. Results

### 3.1. Laying Performance and Feed Cost

**Figure 2:**
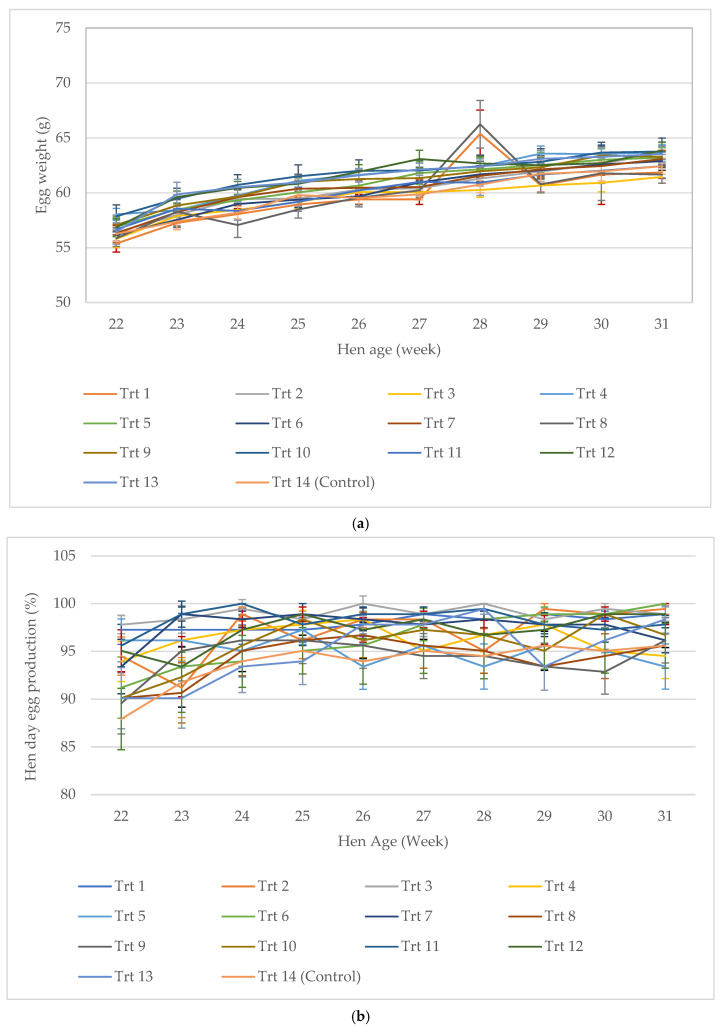

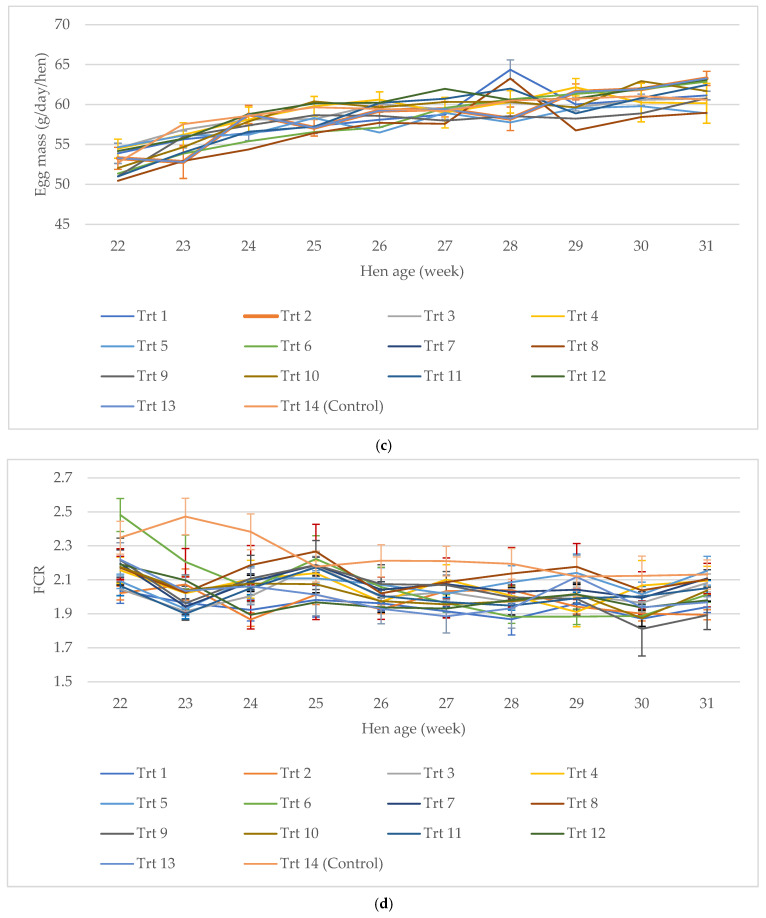
Weekly egg weight (**a**), hen-day egg production (**b**), egg mass (**c**), and FCR (**d**) of the dietary treatments (from 22 to 31 WOA). The dot points represent means, and error bars present standard errors in the means.

The weekly laying performance of hens during the study period (22 to 31 WOA) is presented in Figure 2. This illustration effectively highlights the duration required for hens to stabilize on the treatments and provides an overview of the general performance trends observed. The results showed a slight increasing trend in egg weight with the age of hens (22 to 31 WOA) over the 10 weeks of the study, which subsequently resulted in a similar trend in egg mass. However, the hen-day egg production and FCR remained consistent throughout the study.

The Box–Behnken response surface analysis of feed cost, FCR, and AM/PM intake ratio was performed to select the optimum nutrient levels from different AM/PM diets. Table 8 presents the coefficient estimates and summary statistics of FCR, feed cost, and AM/PM intake ratio in the response surface model. The responses of FCR, feed cost, and AM/PM intake ratio are expressed by the following equations:

FCR: There was a significant response of FCR to AME (apparent metabolizable energy) levels (*p* = 0.019; R^2^ = 0.03):

FCR = 1.91621 − 0.06813 AME^2^.

Feed cost per kg egg mass (FCEM): A similar response was seen for feed cost ($AUD)/kg egg mass (FCEM) to AME levels (*p* = 0.039; R^2^ = 0.03):

FCEM = 0.702909 − 0.023014 AME^2^.

From the above two equations, it can be inferred that the lowest FCR and feed cost can be achieved at either the +1 or −1 AME level. Sensibly, the Pearson correlation showed that FCEM is highly correlated with FCR (*p* < 0.001; R^2^ = 0.959).

AM/PM intake ratio: Looking at all three variables together, there was a significant response of the AM:PM ratio to CP (crude protein), Ca (calcium), and AME levels (*p* = 0.002; R2 = 0.06) in the relationship:

AM/PM ratio = −0.037189 × CP − 0.035632 × Ca × AME + 0.826963.

**Figure 3:**
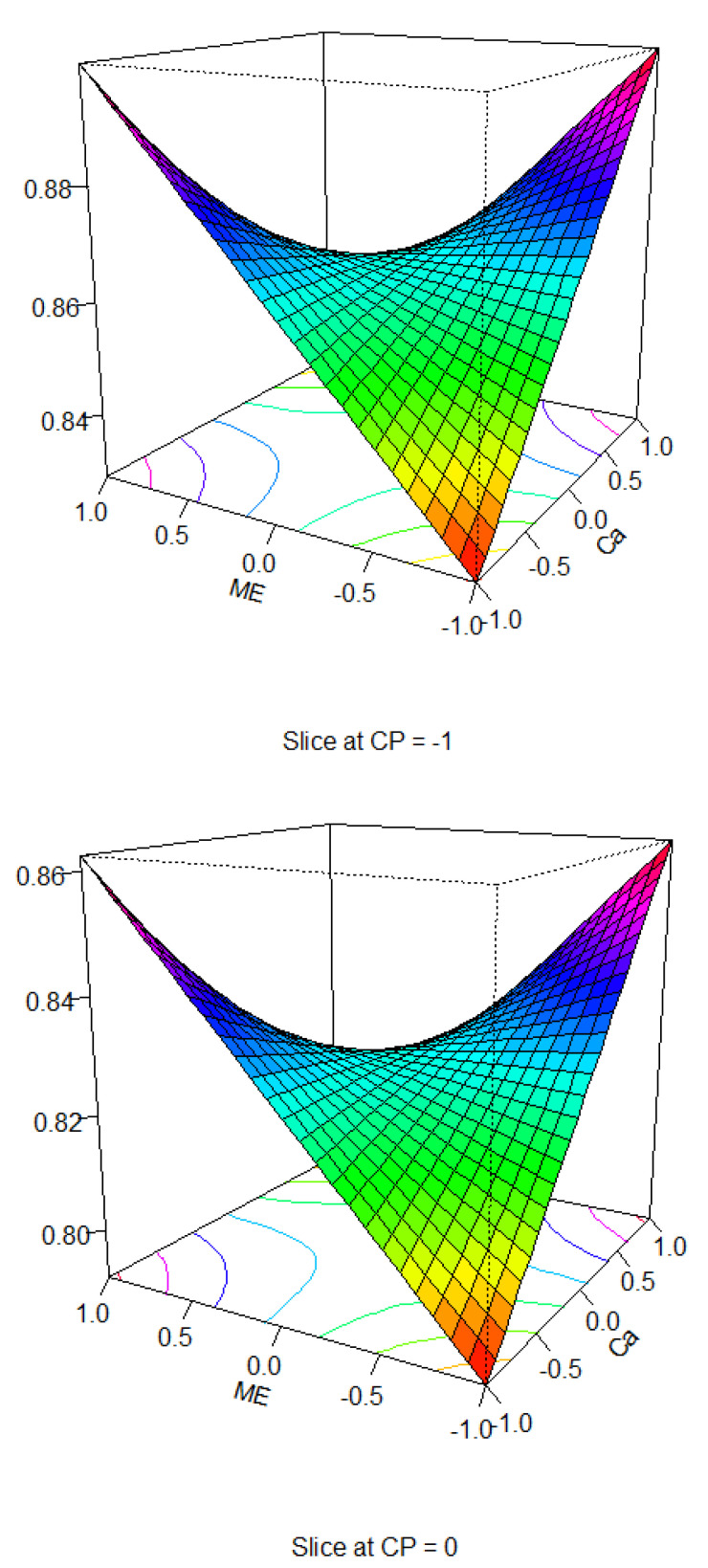

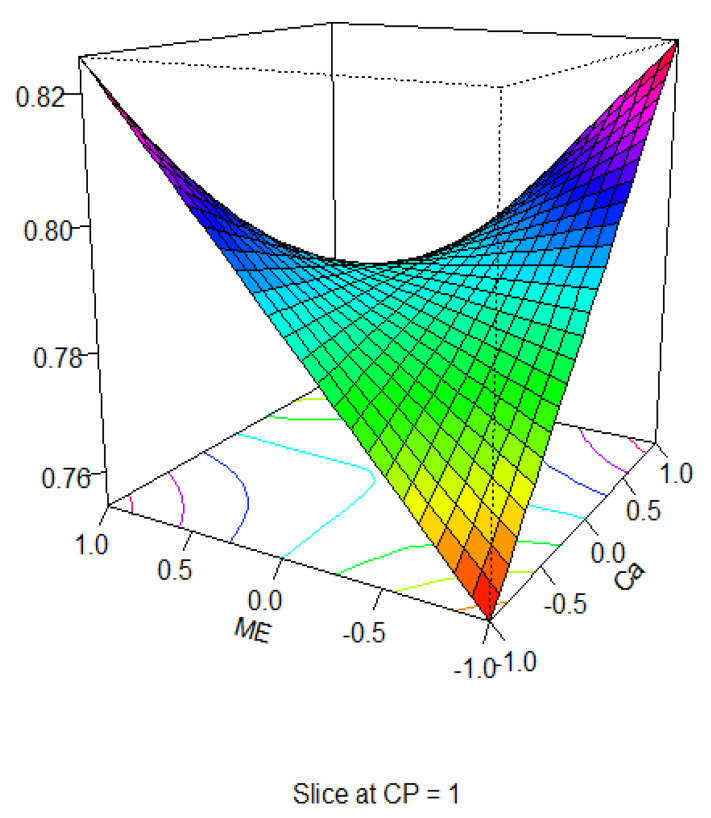
Response surface plots describing the interaction between AM/PM intake ratio, Ca and ME levels at different levels of CP (level −1 CP = AM 19.6%/PM 18.4%, level 0 CP = AM 20.3%/PM 17.7% and level 1 CP = AM 21%/PM 17%).

This relationship is highly significant, but the R square value is small, indicating that there is still a lot of variability unexplained in this model, likely due to the individual dietary selection of each hen. These relationships are represented in the following plots (Figure 3), where we can examine the interaction between AM/PM intake ratio, Ca and AME levels at different levels of CP. Primarily, the important point from this analysis is that hens select between the AM and PM diets, and the degree of diet selection might depend on the composition of the diets themselves. As previously shown, the lowest FCR and feed cost can be achieved with either the 1 or −1 AME level. As the PM feed is cheaper, and the −1 AME level generated the greatest selection toward the PM feed, it is sensible to choose an AME level of −1, coupled with a Ca level of −1.

Optimal AM/PM diet: After evaluating all the relationships, the optimal AM/PM diet chosen has a Ca level of −1 or (3.3/4.9)%, a CP level of 1 or (21/17)%, and an AME level of −1 or (12/11.12) MJ/kg, giving the best combinations of AM/PM as (21/17)% CP, Ca and (12/11.12) MJ/kg. Additionally, it is noteworthy that many of the AMPM diet combinations explored in this cage study compared favorably with the treatment 14 industry control.

Additionally, the results of the overall laying performance, feed and nutrient intake, and AM/PM intake ratio for the entire 10 weeks of study are presented in Table 9 and Table 10. Laying performance indices like egg weight, egg mass, and hen-day egg production were found to be similar between dietary treatments (*p* > 0.05, Table 9). The results of FCR showed that most of the AM/PM treatments performed better than the control treatment (*p* = 0.017, Table 9). Other key performance parameters, including egg weight, hen-day egg production, egg mass, feed intake, and feed cost, did not show significant differences between the AM/PM treatments and the control group (*p* > 0.05, Table 9). However, feed intake results indicated that the birds on the control treatment had a numerically higher feed intake compared to those on the AM/PM treatments (*p* = 0.06, Table 9). This resulted in higher feed costs for the control group, which approached significance (*p* = 0.062, Table 9). Similarly, AM intake showed a trend toward a difference between treatments (*p* = 0.063, Table 10). In contrast, there was a significant difference in PM feed intake between the AM/PM treatment groups (*p* = 0.007, Table 10). Likewise, the total Ca intake and the AM/PM intake ratios for the AM/PM treatments differed significantly (*p* < 0.001, Table 10). However, the total intake of gross energy and CP remain similar between AM/PM treatments (*p* > 0.05, Table 10).

### 3.2. Hen Weight, Weight Gain and Mortality

Table 11 presents the findings on hen weight at weeks 1, 5, and 10, along with weight gain during weeks 1–5, 5–10, and 1–10. Body weight and weight gain at various stages of the trial were comparable across dietary treatments (*p* > 0.05, Table 11). During the whole experimental period, only one bird from treatment 4 died in week 8, and the death was not related to the nutritional intervention of the experiment.

### 3.3. Egg Quality

Results from various measures of internal and external egg quality at week 10 are presented in Table 12 and Table 13. The findings regarding week 10 egg proportion parameters are detailed in Table 14. Internal egg quality metrics such as albumen height, yolk height, yolk diameter, yolk index, and Haugh unit showed no significant differences between treatments (*p* > 0.05, Table 12). In contrast, yolk color scores were significantly higher in most of the AM/PM treatments compared to the control treatment (*p* = 0.002, Table 12). Treatments 7 and 13 performed best in terms of yolk color score (Table 12). Additionally, external egg quality factors, including egg length, width, shape index, reflectivity, shell thickness, and shell breaking strength, were comparable among the experimental treatments (*p* > 0.05, Table 13). Likewise, the weight and percentage proportions of albumen, yolk, and shell did not vary significantly across treatments (*p* > 0.05, Table 14).

### 3.4. Serum Ca Level

The outcomes of serum Ca analysis at week 10 are illustrated in Table 15. Serum Ca levels of hens were found to be similar among all dietary treatments (*p* = 0.238, Table 15).

### 3.5. Apparent Digestibility of Nutrients

Table 16 presents the findings on the apparent digestibility of key nutrients, including dry matter (DM), energy, protein, calcium (Ca), and phosphorus (P) at week 10. The analysis revealed that the digestibility percentages for DM, energy, and P were comparable across treatments (*p* > 0.05, Table 16). However, the digestibility of protein and Ca were statistically different among some of the treatments (*p* < 0.05, Table 16). Table 16 demonstrates that hens fed treatments 10 and 4 had the highest apparent protein and Ca digestibility, respectively, compared to the control treatment (*p* < 0.05).

## 4. Discussion

Reducing feed costs is critical in poultry production as feed accounts for approximately 60–70% of the total production expenses, making it the most significant factor influencing profitability [3,13]. Efficient feed utilization not only enhances economic returns but also supports sustainability by minimizing resource wastage and environmental impact. Strategies such as formulating balanced diets based on the precise nutrient requirements of hens, incorporating alternative feed ingredients, and employing feed management techniques like phase feeding or AM/PM feeding have proven effective in reducing costs [2,3,41,42,43]. Moreover, optimizing feed efficiency directly correlates with improved production performance and profitability, especially in the context of rising feed ingredient prices globally [2,44,45]. So, scientists are striving to implement innovative nutritional strategies to obtain better feed efficiency while maintaining hen’s health and productivity. The results of the current study indicate that most AM/PM feeding regimens outperformed the control diet in terms of feed conversion ratio (FCR) and tended to have lower feed intake and thus decreased feed cost. Hens receiving AM/PM treatments generally exhibited a lower FCR compared to those on the control diet, primarily due to reduced feed consumption. This reduction in feed intake directly translated into decreased feed costs for the AM/PM treatment groups, highlighting the economic advantage of this feeding strategy. Studies have reported that such tailored feeding regimens lower FCR due to optimized nutrient partitioning, which supports production efficiency without overfeeding specific nutrients at inappropriate times [12,18]. Moreover, the reduced feed intake observed in AM/PM feeding systems contributes to the lower FCR, as hens consume only what is needed for their specific physiological demands during each phase of the day [14]. These studies collectively suggest that adjusting the protein, energy, and calcium content in diets at different times of the day can lead to improved feed efficiency and overall productivity in laying hens. These improvements in FCR are economically significant, as feed costs account for a major portion of production expenses in commercial layer operations. The AM/PM intake ratio in laying hens reflects the distribution of feed consumption between morning (AM) and afternoon/evening (PM) periods. This ratio is critical as it mirrors the hens’ physiological requirements and feeding behavior, both of which are influenced by their reproductive cycles and metabolic needs. Hens have an innate ability to choose feeds that meet their physiological requirements, particularly during specific stages of production when they have options [46]. They demonstrate selective feeding behavior when presented with diets differing in nutrient composition, enabling them to balance their nutrient intake when offered diverse feed options [14]. This ability to select feeds based on nutrient content is especially pronounced in systems where hens are provided with AM/PM diets [4]. The findings of the present study also revealed that hens exhibited distinct feed selection behaviors between the AM and PM diets. Specifically, hens consumed more of the PM diets, which consequently led to a higher intake of Ca. Moreover, the PM diets were more cost-effective than the AM diets due to their reduced reliance on expensive protein ingredients. This difference in feed composition contributed to a lower overall feed cost in the AM/PM feeding treatments compared to the control group that offered a single, conventional diet throughout the day.

The comparative results of egg weight, egg mass, and hen-day egg production in the present study remain similar between dietary treatments. This might support other studies [25,29,47] where both the split and conventional feeding showed a similar impact on egg production. However, the improved effect was seen in other trials that showed a tendency to increase egg production [18,20] and egg mass [18] in hens fed AM/PM feed. On the other hand, another study conducted by Lee and Ohh [29] observed decreased egg weight, which contradicts the findings of the present study. However, the results of the present study also suggest that the test treatments did not negatively impact egg production, as the production percentage (95–99%) closely matched the standard performance benchmarks for Hy-Line Brown hens. This indicates that the dietary treatments were effective in maintaining optimal productivity, comparable to the expected standards outlined in the Hy-Line Brown performance guidelines [48]. This consistency reinforces the viability of the test treatments for sustaining egg production without compromising hen efficiency or health.

In the present study, the hens’ body weights did not differ between treatments. This finding was supported by El-Kelawy [18] and Traineau et al. [19], who observed that hens that were fed a higher protein/energy diet in the morning and a lower protein/energy, higher calcium diet in the afternoon had improved feed conversion ratios (FCR), but their body weights were not negatively impacted. This effect could be attributed to the absence of excess energy consumption during the evening feeding period, which minimizes fat deposition. Overconsumption of energy, particularly during the later hours of the day, can lead to an increased risk of overweight hens and fatty liver syndrome—issues that are prevalent in commercial laying hen operations [5,49,50]. This suggests that AM/PM feeding strategies may optimize feed intake and energy utilization without causing detrimental effects on hen body weight. However, a longer experiment might reveal a noticeable difference.

Like the present study, the effect of split feeding on the external and internal quality of eggs in laying hens has been a focus of several studies, primarily due to its potential to optimize nutrient utilization. Studies also show that this feeding strategy improves egg shell quality due to the timing of calcium intake, as calcium is required for shell formation in the later stages of the day [51] when hens are less likely to experience excess protein [14,15]. The results of the present study revealed that the AM/PM feeding strategy was found to have no impact on any of the external and internal egg quality parameters except the yolk color score. In contrast, An et al. [52] found better shell strength and thickness with increased levels of Ca in the diet. Similarly, a study by Saki et al. [51] supports the beneficial effect of a higher level of coarse Ca in the evening on egg shell thickness and weight. Another study conducted by El-Kelawy [18] demonstrated improved egg quality in terms of egg length and width and albumen weight and height, and Haugh unit in hens fed an AM/PM diet compared to those on a standard continuous diet. However, studies by other poultry nutritionists [17,20,53,54] did not find any improved effect of split feeding on most of the egg quality parameters. These findings partially support the result of the present study. The improved yolk color in the present study may be attributed to the efficient utilization of nutrients under the AM/PM feeding regimen.

AM/PM feeding schedules are designed to align calcium availability with the physiological needs of hens, particularly during eggshell formation, which predominantly occurs in the afternoon and evening. In the present study, serum calcium (Ca) levels were analyzed to determine if the reduced dietary Ca in the morning had any adverse effects. The findings revealed no significant differences in serum Ca levels between hens of test treatments fed AM/PM diets and those on conventional continuous diets. This suggests that the AM/PM feeding strategy effectively maintains serum calcium homeostasis. This result is consistent with previous research by An et al. [52], who found stable serum calcium levels in hens under AM/PM feeding regimens. Similarly, Saki et al. [51] observed no significant changes in plasma calcium levels despite varying dietary calcium concentrations provided at different times of the day. These studies collectively indicate that AM/PM feeding schedules, with lower morning and higher evening calcium levels, do not compromise calcium metabolism and are compatible with maintaining hens’ health and eggshell quality.

The findings of the present study revealed that most of the AM/PM treatments significantly improved the apparent digestibility of protein and calcium compared to the control diet. Previous studies discovered that excess Ca in the diet significantly reduces nutrient digestibility and feed efficiency in poultry [55]. In the study, hens on the AM/PM treatments did not consume excess calcium in the morning, which may have facilitated better protein digestion in most of the test treatments compared to those on the control diet, where excess dietary calcium could hinder protein digestibility [5]. Furthermore, the improved calcium digestibility in the AM/PM treatments of the present study may be attributed to the higher dietary calcium provided in the afternoon/evening, aligning with the hen’s physiological requirements for eggshell and membrane formation. This timing ensures optimal calcium absorption and utilization when the demand for calcium is at its peak for egg production, enhancing overall digestibility and efficiency compared to the control diet, where calcium levels are constant throughout the day. However, DM, energy, and P digestibility remain unchanged between treatments of the present study, indicating that these nutrients were absorbed similarly regardless of the dietary variation, and AM/PM diets had no negative impact on the utilization of these nutrients. This suggests that the way hens processed these nutrients varied, potentially influencing their overall nutritional intake and health. The distinction in protein and calcium digestibility may have important implications for optimizing diets to enhance hen performance and egg quality, highlighting the need for further investigation into dietary formulations.

## 5. Conclusions

In conclusion, the findings of the present study highlight the advantages of AM/PM feeding regimens over conventional single diets in laying hens under commercial conditions. AM/PM feeding demonstrated improved feed efficiency, enhanced yolk color, and better protein and calcium digestibility, alongside reduced feed costs. These improvements underscore the potential of this feeding strategy to enhance nutrient utilization and align dietary provisions with the physiological requirements of hens throughout the day. The practical benefits of this approach are that it provides a cost-effective and nutritionally efficient solution, directly supporting the productivity and sustainability of the poultry industry. However, the current findings also point to the need for further investigations to refine and assess the identified optimal levels of nutrients (energy, protein, and calcium) in AM/PM diets. Such research would provide a more comprehensive understanding of this strategy’s potential and its application in diverse poultry production systems.

## Figures and Tables

**Table 1:** Factor description for the study.

Factor	Level (−1)	Level (0)	Level (1)
(1) Ca (%)	AM 3.3/PM 4.9	AM 2.5/PM 5.7	AM 1.6/PM 6.6
(2) CP (%)	AM 19.6/PM 18.4	AM 20.3/PM 17.7	AM 21.0/PM 17.0
(3) AME (MJ/kg)	AM 12.0/PM 11.2	AM 12.4/PM 10.8	AM 12.8/PM 10.4

**Table 2:** The design matrix of the study.

Treatment	Factor 1 Level	Factor 2 Level	Factor 3 Level
1	−1	−1	0
2	−1	0	−1
3	−1	0	1
4	−1	1	0
5	0	−1	−1
6	0	−1	1
7	0	0	0
8	0	1	−1
9	0	1	1
10	1	−1	0
11	1	0	−1
12	1	0	1
13	1	1	0

**Table 3:** Schedule of dietary treatments for the study.

Treatment	Factor 1 Ca (%)	Factor 2 CP (%)	Factor 3 AME (MJ/kg)
1	AM 3.3/PM 4.9	AM 19.6/PM 18.4	AM 12.4/PM 10.8
2	AM 3.3/PM 4.9	AM 20.3/PM 17.7	AM 12.0/PM 11.2
3	AM 3.3/PM 4.9	AM 20.3/PM 17.7	AM 12.8/PM 10.4
4	AM 3.3/PM 4.9	AM 21.0/PM 17.0	AM 12.4/PM 10.8
5	AM 2.5/PM 5.7	AM 19.6/PM 18.4	AM 12.0/PM 11.2
6	AM 2.5/PM 5.7	AM 19.6/PM 18.4	AM 12.8/PM 10.4
7	AM 2.5/PM 5.7	AM 20.3/PM 17.7	AM 12.4/PM 10.8
8	AM 2.5/PM 5.7	AM 21.0/PM 17.0	AM 12.0/PM 11.2
9	AM 2.5/PM 5.7	AM 21.0/PM 17.0	AM 12.8/PM 10.4
10	AM 1.6/PM 6.6	AM 19.6/PM 18.4	AM 12.4/PM 10.8
11	AM 1.6/PM 6.6	AM 20.3/PM 17.7	AM 12.0/PM 11.2
12	AM 1.6/PM 6.6	AM 20.3/PM 17.7	AM 12.8/PM 10.4
13	AM 1.6/PM 6.6	AM 21.0/PM 17.0	AM 12.4/PM 10.8
14	4.1	19	11.63

**Table 4:** Composition of the experimental treatments.

Ingredient (g/kg)	T14	T1AM	T1PM	T2AM	T2PM	T3AM	T3PM	T4AM	T4PM	T5AM	T5PM	T6AM	T6PM	T7AM
Soybean meal	122.9	150.5	139.7	160	122.6	166	155.3	174.9	128.5	141.3	142.1	147.1	147	156
Canola oil	37.2	56.4	37.6	43.6	36.5	71.7	40	59.2	37.6	31.2	50.2	59	39.9	46.4
Barley	100	100	100	100	100	100	100	100	100	100	100	100	100	100
Wheat	533.5	509.5	465.7	512.8	508.4	478.7	417.6	482.2	472.6	564.5	452	531.3	424.2	535
Canola meal	100	100	100	100	100	100	100	100	100	100	100	100	100	100
^1^ Limestone flour	47.89	38.11	58.77	38.08	58.83	38.08	58.71	38.04	58.79	27.53	68.5	27.58	68.47	27.54
^2^ Limestone grit	47.9	38.1	58.77	38.07	58.83	38.07	58.71	38.05	58.79	27.53	68.49	27.57	68.46	27.55
Salt	1.54	1.51	1.57	1.49	1.53	1.54	1.49	1.53	1.67	1.42	1.6	1.47	1.65	1.46
Monocal phos	3.63	0	7.9	0	7.77	0	8.09	0	7.97	0.2	11.49	0	11.64	0
Sodium bicarb	1.13	1.2	1.2	1.21	1.2	1.2	1.76	1.21	1.05	1.6	1.18	1.22	1.16	1.23
L-lysine HCl	0.92	0.96	0.97	0.98	1.05	0.89	0.27	0.89	0.67	1.13	0.89	1.05	0.82	1.04
DL-methionine	1.68	1.81	1.68	1.91	1.48	1.97	1.48	2.08	1.35	1.69	1.71	1.76	1.77	1.86
L-threonine	0.14	0.24	0.21	0.25	0.2	0.25	0	0.26	0.04	0.26	0.21	0.25	0.2	0.26
Bentonite	0	0	24.33	0	0	0	55	0	29.29	0	0	0	33.08	0
^3^ Vit + min premix	1	1	1	1	1	1	1	1	1	1	1	1	1	1
^4^ Pigment red	0.04	0.04	0.04	0.04	0.04	0.04	0.04	0.04	0.04	0.04	0.04	0.04	0.04	0.04
^5^ Pigment yellow	0.03	0.03	0.03	0.03	0.03	0.03	0.03	0.03	0.03	0.03	0.03	0.03	0.03	0.03
^6^ Xylanase	0.1	0.1	0.1	0.1	0.1	0.1	0.1	0.1	0.1	0.1	0.1	0.1	0.1	0.1
^7^ Phytase	0.1	0.1	0.1	0.1	0.1	0.1	0.1	0.1	0.1	0.1	0.1	0.1	0.1	0.1

^1^ Limestone 38 Flour: Attunga AGLime, Ca 38.4% (96% as calcium carbonate), Neutralizing value 97.5%, Fineness minimum 95% (passing 0.71 mm sieve) and 55% fines (passing 0.25 mm sieve), Graymont (Australia) Pty. Ltd., North Sydney, NSW, Australia; ^2^ Limestone 38 Grit: Poultry Grit, Minimum Ca as calcium carbonate 39%, Minimun neutralizing value 98%, Sizing 3.5 mm to 1000 microns, Australian Agricultural Mineral (AAM), Gore, QLD, Australia; ^3^ Vitamin–mineral premix included the following per kg of diet: 10,000 IU of vitamin A, 3000 IU of vitamin D, 20 mg of vitamin E, 3 mg of vitamin K, 35 mg of nicotinic acid (niacin), 12 mg of pantothenic acid, 1 mg of folic acid, 6 mg of riboflavin (B2), 0.02 mg of cyanocobalamin (B12), 0.1 mg of biotin, 5 mg of pyridoxine (B6), 2 mg of thiamine (B1), 8 mg of copper as copper sulfate pentahydrate, 0.2 mg of cobalt as cobalt sulfate 21%, 0.5 mg of molybdenum as sodium molybdate, 1 mg of iodine as potassium iodide 68%, 0.3 mg of selenium as selenium 2%, 60 mg of iron as iron sulfate 30%, 60 mg of zinc as zinc sulfate 35%, 90 mg of manganese as manganous oxide 60%, and 20 mg of antioxidant. ^4^ Pigment red (Jabiru red): Canthaxanthin 10%, Guangzhou Juyuan Bio-Chem Co., Ltd., Guangzhou, China; ^5^ Pigment yellow (Jabiru yellow): Apocarotenoic acid ethyl ester 10%, Guangzhou Juyuan Bio-Chem Co., Ltd., Guangzhou, China; ^6^ Xylanase: Axtra XB TPT 201, Danisco Animal Nutrition (IFF), Oegstgeest, The Netherlands; ^7^ Phytase: Axtra PHY Gold, Danisco Animal Nutrition (IFF), Oegstgeest, The Netherlands.

**Table 5:** Composition of the experimental treatments continued.

Ingredient (g/kg)	T7PM	T8AM	T8PM	T9AM	T9PM	T10AM	T10PM	T11AM	T11PM	T12AM	T12PM	T13AM	T13PM
Soybean meal	129	164.9	129.6	170.9	136.1	137.2	146.7	152.4	138.2	152.1	136.1	161	134.6
Canola oil	37.6	33.7	48.4	61.4	39.8	33.8	48.4	31.3	61.2	49.1	39.8	36.5	46.6
Barley	100	100	100	100	100	100	100	100	100	100	100	100	100
Wheat	472.3	538.8	467	505.1	432.9	587.6	426.2	555.6	421.8	557.3	432.9	561	440.7
Canola meal	100	100	100	100	100	100	100	100	100	100	100	100	100
Limestone flour	68.53	27.52	68.52	27.52	68.49	17.05	78.21	26.59	78.21	17.02	78.22	16.98	78.23
Limestone grit	68.52	27.51	68.52	27.51	68.48	17.04	78.2	26.6	78.21	17.01	78.22	16.99	78.23
Salt	1.58	1.44	1.68	1.49	1.74	1.39	1.64	1.43	1.66	1.42	1.64	1.41	1.72
Monocal phos	11.49	0	11.52	0	11.69	0	15.15	0	15.27	0	15.21	0	15.17
Sodium bicarb	1.18	1.23	1.05	1.22	1.03	1.24	1.16	1.23	1.16	1.23	1.16	1.24	1.03
L-lysine HCl	0.97	1.02	0.66	0.94	0.56	1.18	0.83	1.08	0.83	1.09	0.86	1.08	0.58
DL-methionine	4.9	1.96	1.36	2.03	1.42	1.64	1.77	1.82	1.66	1.82	1.64	1.91	1.41
L-threonine	0.2	0.27	0.04	0.27	0.02	0.26	0.2	0.26	0.19	0.26	0.19	0.28	0.02
Bentonite	2.15	0	0	0	36.14	0	0	0	0	0	12.41	0	0
Vit + min premix	1	1	1	1	1	1	1	1	1	1	1	1	1
Pigment red	0.04	0.04	0.04	0.04	0.04	0.04	0.04	0.04	0.04	0.04	0.04	0.04	0.04
Pigment yellow	0.03	0.03	0.03	0.03	0.03	0.03	0.03	0.03	0.03	0.03	0.03	0.03	0.03
Xylanase	0.1	0.1	0.1	0.1	0.1	0.1	0.1	0.1	0.1	0.1	0.1	0.1	0.1
Phytase	0.1	0.1	0.1	0.1	0.1	0.1	0.1	0.1	0.1	0.1	0.1	0.1	0.1

**Table 6:** Calculated nutrient value of the experimental treatments in the study.

Dietary Treatment		Dry Matter, %	AME, MJ/kg	CP, %	Dig. Lys, %	Ca, %	P, %	Na, g/kg
1	AM	91.5	12.11	18.2	0.810	3.2	0.352	0.160
	PM	91.6	10.75	17.2	0.770	4.9	0.539	0.160
2	AM	91.4	11.76	18.7	0.387	3.2	0.355	0.160
	PM	91.6	11.08	16.9	0.743	4.9	0.539	0.160
3	AM	91.5	12.45	18.6	0.837	3.2	0.351	0.160
	PM	91.5	10.39	17.3	0.743	4.9	0.539	0.160
4	AM	91.4	12.11	19.1	0.861	3.2	0.354	0.160
	PM	91.5	10.73	16.7	0.719	4.9	0.539	0.160
5	AM	91.1	11.76	18.4	0.814	2.4	0.365	0.160
	PM	91.8	11.07	17.1	0.766	5.7	0.627	0.160
6	AM	91.3	12.44	18.3	0.814	2.4	0.355	0.160
	PM	91.7	10.39	17.1	0.766	5.7	0.627	0.160
7	AM	91.2	12.10	18.8	0.837	2.4	0.358	0.160
	PM	91.8	10.73	16.9	0.743	5.7	0.627	0.160
8	AM	91.1	11.76	19.3	0.861	2.4	0.361	0.160
	PM	91.8	11.07	16.7	0.719	5.7	0.627	0.160
9	AM	91.3	12.44	19.1	0.861	2.4	0.357	0.160
	PM	91.7	10.39	16.6	0.719	5.7	0.627	0.160
10	AM	91.0	12.10	18.5	0.814	1.6	0.363	0.160
	PM	92.0	10.73	17.1	0.766	6.5	0.715	0.160
11	AM	91.1	11.76	18.8	0.837	2.3	0.361	0.160
	PM	92.1	11.07	16.6	0.743	6.5	0.715	0.160
12	AM	91.1	12.44	18.9	0.837	1.6	0.361	0.160
	PM	91.9	10.39	16.6	0.743	6.5	0.715	0.160
13	AM	91.0	12.10	19.3	0.861	1.6	0.364	0.160
	PM	92.0	10.73	16.6	0.719	6.5	0.715	0.160
Control		91.5	11.420	17.2	0.740	4.0	0.440	0.160

**Table 7:** Analyzed nutrient value of the experimental treatments in the study.

Dietary Treatment		Dry Matter, %	Gross Energy, Kcal/kg	CP, %	Ca, %	P, %	Na, g/kg
1	AM	91.27	3956	19.13	3.57	0.49	1.51
	PM	91.74	3570	17.60	5.54	0.64	1.42
2	AM	91.52	3938	19.40	3.60	0.50	1.25
	PM	91.86	3660	17.61	5.47	0.62	1.16
3	AM	91.50	4050	18.88	3.52	0.49	1.05
	PM	91.81	3438	18.15	5.88	0.64	1.81
4	AM	91.13	3982	19.23	3.65	0.48	1.27
	PM	91.68	3553	17.22	5.19	0.61	1.46
5	AM	90.92	3919	19.75	2.63	0.51	1.11
	PM	92.22	3668	17.82	5.83	0.75	1.11
6	AM	91.26	4090	18.44	2.42	0.49	1.26
	PM	92.03	3411	17.98	6.36	0.71	1.41
7	AM	91.24	4053	19.72	2.49	0.50	1.12
	PM	92.26	3542	16.95	6.44	0.70	1.32
8	AM	91.05	3982	19.87	2.46	0.49	1.01
	PM	92.17	3631	16.82	6.29	0.77	1.57
9	AM	91.13	4088	19.89	2.40	0.50	1.17
	PM	91.95	3439	16.64	6.29	0.68	1.68
10	AM	90.92	4023	19.44	1.52	0.51	1.08
	PM	92.08	3531	17.39	6.68	0.86	1.57
11	AM	90.78	3933	19.09	2.62	0.51	1.04
	PM	92.44	3606	17.26	6.70	0.82	1.35
12	AM	91.30	4136	18.99	1.75	0.50	1.23
	PM	92.32	3412	16.92	7.11	0.89	1.23
13	AM	91.25	4095	19.84	1.44	0.51	1.18
	PM	92.47	3535	17.54	6.69	0.83	1.24
Control		91.48	3788	17.57	4.29	0.53	1.25

**Table 8:** Coefficient estimates and summary statistics of FCR, FCEM, and AM/PM intake ratio in response to different levels of Ca (X_1_), CP (X_2_), and AME (X_3_).

	FCR		FCEM		AM/PM Intake Ratio	
Variables ^1^	Coefficient	*p*-Value	Coefficient	*p*-Value	Coefficient	*p*-Value
First order						
X_1_	-	-	-	-	-	-
X_2_	-	-	-	-	−0.037	0.004
X_3_	-	-	-	-	-	-
Second order						
X_1_	-	-	-	-	-	-
X_2_	-	-	-	-	-	-
X_3_	−0.068	0.019	−0.023	0.039	-	-
Interactions						
X_1_:X_2_	-	-	-	-	-	-
X_2_:X_3_	-	-	-	-	-	-
X_1_:X_3_	-	-	-	-	−0.036	0.047
Intercept	1.916	<0.001	0.703	<0.001	0.827	<0.001
R^2^	0.033		0.026		0.075	
R^2^_adj_	0.027		0.020		0.063	
*p*-value		0.019		0.039		0.002

^1^ X_1_ = calcium factor, X_2_ = crude protein factor, X_3_ = AME factor.

**Table 9:** Laying performance of hens on different dietary treatments over 10 weeks of the study.

Treatment	Egg Weight (g)	Hen-Day Egg Production (%)	Egg Mass (g)	Feed Intake (g)	FCR (kg Feed/kg Egg)	Feed Cost (AUD/Bird/Day)	Feed Cost (AUD/kg Egg Mass)
1	59.8	98.0	58.6	115	1.962 ^a^	0.042	0.718
2	60.4	97.0	58.6	117	1.995 ^a^	0.042	0.720
3	59.5	99.0	58.8	118	2.010 ^a^	0.044	0.749
4	61.4	97.0	59.6	121	2.037 ^ab^	0.045	0.733
5	60.5	98.1	59.3	120	2.016 ^ab^	0.044	0.733
6	60.3	98.1	59.1	119	2.008 ^ab^	0.044	0.738
7	60.5	97.6	59.0	123	2.077 ^ab^	0.045	0.766
8	60.3	97.6	58.9	118	2.005 ^a^	0.043	0.729
9	61.1	98.2	59.9	120	2.003 ^a^	0.044	0.739
10	61.7	95.7	59.0	119	2.026 ^ab^	0.043	0.737
11	60.1	98.2	59.0	119	2.021 ^ab^	0.044	0.743
12	61.5	97.2	59.7	119	1.994 ^a^	0.043	0.727
13	61.4	96.1	59.0	121	2.045 ^ab^	0.044	0.744
14	59.8	97.4	58.3	127	2.182 ^b^	0.045	0.776
SEM	0.20	0.20	0.20	0.59	0.010	0.0002	0.004
*p*-value	0.532	0.195	0.982	0.060	0.017	0.133	0.062

^a,b^ Means within columns not sharing a common suffix are significantly different at the 5% level of probability. Here, treatment 14 was not a part of the response surface analysis; it was only added to compare the results with other treatments.

**Table 10:** Feed and nutrient intake of hens on AM/PM treatment over 10 weeks of the study.

Treatment	AM Feed Intake (g/Hen/Day)	PM Feed Intake (g/Hen/Day)	Total Energy Intake (Kcal/Hen/Day)	Total CP Intake (g/Hen/Day)	Total Ca Intake (g/Hen/Day)	AM/PM Intake Ratio
1	52.7	62.1 ^a^	430.36	21.02	5.32	0.860 ^b^
2	52.4	64.4 ^ab^	442.05	21.51	5.41	0.818 ^ab^
3	54.4	63.7 ^ab^	439.36	21.83	5.66	0.861 ^b^
4	55.8	65.4 ^ab^	454.40	21.98	5.43	0.856 ^b^
5	50.9	68.8 ^ab^	445.66	22.01	5.29	0.743 ^ab^
6	54.0	64.8 ^ab^	441.46	21.58	5.41	0.840 ^b^
7	54.4	68.1 ^ab^	461.98	22.28	5.74	0.804 ^ab^
8	51.8	66.3 ^ab^	443.92	21.30	5.40	0.783 ^ab^
9	55.2	64.6 ^ab^	439.62	21.33	5.29	0.864 ^b^
10	52.7	66.4 ^ab^	446.76	21.81	5.24	0.800 ^ab^
11	48.7	70.5 ^b^	445.62	21.46	6.00	0.693 ^a^
12	53.7	65.3 ^ab^	445.00	21.25	5.59	0.829 ^ab^
13	51.8	68.8 ^ab^	449.83	22.07	5.28	0.757 ^ab^
SEM	0.42	0.45	8.21	0.40	0.11	0.009
*p*-value	0.063	0.007	0.581	0.548	<0.001	<0.001

^a,b^ Means within columns not sharing a common suffix are significantly different at the 5% level of probability.

**Table 11:** Hen weights of the dietary treatments over 10 weeks of the study.

Treatment	Hen Weight (g)			Weight Gain (g)		
	Week 1	Week 5	Week 10	Week 1–5	Week 5–10	Week 1–10
1	1910	2027	2142	117	115	232
2	1963	2075	2197	112	123	235
3	1921	2061	2173	140	112	252
4	1973	2107	2203	135	96	230
5	1942	2078	2195	136	116	252
6	1940	2087	2190	147	103	250
7	1974	2143	2273	169	130	299
8	1963	2095	2222	132	127	259
9	1961	2092	2213	131	122	253
10	1939	2067	2179	128	112	240
11	1925	2065	2185	140	119	259
12	1937	2067	2178	130	111	241
13	1982	2107	2217	125	109	234
14	1932	2085	2191	154	106	259
SEM	8.41	9.95	10.98	4.15	3.13	5.23
*p*-value	0.936	0.906	0.905	0.570	0.822	0.629

Here, treatment 14 was not a part of the response surface analysis; it was only added to compare the results with other treatments.

**Table 12:** Internal egg quality of hens on different dietary treatments at week 10 of the study.

Treatment	Albumen Height (mm)	Yolk Color	Haugh Unit	Yolk Height (mm)	Yolk Diameter (mm)	Yolk Index
1	10.30	11.92 ^ab^	99.12	23.28	33.70	0.706
2	10.96	12.75 ^bcd^	102.08	23.89	32.77	0.741
3	11.10	12.33 ^abc^	102.70	23.38	32.61	0.733
4	10.83	12.77 ^bcd^	101.63	23.36	32.68	0.728
5	9.72	12.46 ^abc^	99.45	24.18	35.02	0.711
6	10.75	12.31 ^abc^	101.63	23.43	31.75	0.749
7	10.58	13.62 ^d^	100.35	23.98	33.25	0.736
8	9.78	12.92 ^cd^	97.59	23.24	32.38	0.728
9	11.65	12.27 ^abc^	104.61	23.70	30.58	0.784
10	10.42	13.15 ^cd^	100.00	23.65	33.85	0.709
11	9.47	13.15 ^cd^	95.24	23.22	34.86	0.685
12	10.40	12.77 ^bcd^	101.99	23.65	35.01	0.688
13	11.55	13.46 ^d^	104.14	23.50	34.13	0.702
14	10.78	11.69 ^a^	101.40	23.65	32.46	0.734
SEM	0.17	0.10	0.68	0.10	0.36	0.008
*p*-value	0.436	0.002	0.498	0.856	0.545	0.720

^a,b,c,d^ Means within columns not sharing a common suffix are significantly different at the 5% level of probability. Here, treatment 14 was not a part of the response surface analysis; it was only added to compare the results with other treatments.

**Table 13:** External egg quality of hens on different dietary treatments at week 10 of the study.

Treatment	Shell Breaking Strength (Kgf)	Shell Thickness (mm)	Egg Length (mm)	Egg Width (mm)	Egg Shape Index	Reflectivity (%)
1	5.23	0.443	56.7	43.8	0.774	25.1
2	4.64	0.430	57.3	44.0	0.769	25.0
3	5.02	0.438	56.4	44.2	0.784	24.7
4	4.97	0.448	56.5	44.2	0.782	24.4
5	4.87	0.439	57.1	44.3	0.776	23.5
6	4.70	0.436	57.1	44.1	0.773	25.3
7	4.77	0.449	56.8	44.3	0.780	24.2
8	5.12	0.441	56.4	43.7	0.775	24.2
9	4.70	0.432	56.9	44.1	0.776	24.2
10	5.09	0.439	56.7	44.3	0.781	25.1
11	5.16	0.438	56.7	43.7	0.772	24.4
12	4.60	0.434	57.7	44.3	0.769	24.0
13	4.83	0.440	56.7	44.1	0.778	23.6
14	4.88	0.428	56.7	44.1	0.778	24.9
SEM	0.06	0.002	0.11	0.08	0.002	0.22
*p*-value	0.584	0.420	0.745	0.941	0.851	0.951

Here, treatment 14 was not a part of the response surface analysis; it was only added to compare the results with other treatments.

**Table 14:** Egg proportion of hens on different dietary treatments at week 10 of the study.

Treatment	Albumen Weight (g)	Yolk Weight (g)	Shell Weight (g)	Albumen (%)	Yolk (%)	Shell (%)
1	40.28	14.98	6.09	65.63	24.43	9.93
2	41.15	15.51	5.96	65.62	24.83	9.54
3	40.31	15.85	6.20	64.61	25.45	9.94
4	40.67	15.31	6.16	65.36	24.73	9.92
5	41.48	15.72	6.21	65.24	24.93	9.83
6	40.57	15.59	6.06	65.21	25.06	9.72
7	41.63	15.76	6.25	65.38	24.78	9.84
8	40.02	15.18	6.10	65.17	24.85	9.98
9	40.95	15.62	6.07	65.29	25.01	9.70
10	41.51	15.73	6.14	65.45	24.87	9.68
11	40.64	15.41	6.10	65.25	24.91	9.84
12	42.74	15.52	6.09	66.41	24.13	9.46
13	40.41	15.57	6.04	65.15	25.11	9.74
14	40.53	15.97	5.83	65.00	25.62	9.39
SEM	0.27	0.10	0.03	0.18	0.16	0.04
*p*-value	0.913	0.923	0.716	0.938	0.956	0.483

Here, treatment 14 was not a part of the response surface analysis; it was only added to compare the results with other treatments.

**Table 15:** Serum calcium level of hens on different dietary treatments at week 10 of the study.

Treatment	Serum Ca Level (mg/dL)
1	29.04
2	27.82
3	27.14
4	25.29
5	26.10
6	28.28
7	25.81
8	25.35
9	29.98
10	25.05
11	28.25
12	28.80
13	26.53
14	26.23
SEM	1.34
*p*-value	0.238

Here, treatment 14 was not a part of the response surface analysis; it was only added to compare the results with other treatments.

**Table 16:** Apparent nutrient digestibility (%) of hens on different dietary treatments at week 10 of the study.

Treatment	Dry Matter Digestibility	Energy Digestibility	Protein Digestibility	Ca Digestibility	P Digestibility
1	71.42	78.63	46.28 ^bcd^	58.59 ^bc^	28.73
2	67.90	74.39	36.793 ^ab^	53.50 ^bc^	22.24
3	66.82	75.26	46.24 ^bcd^	56.45 ^bc^	27.83
4	71.15	77.20	44.94 ^bcd^	62.13 ^c^	32.24
5	68.76	76.60	38.76 ^abc^	50.67 ^bc^	36.32
6	68.51	75.33	41.35 ^abc^	55.77 ^bc^	39.13
7	68.24	75.51	38.38 ^abc^	52.28 ^bc^	28.83
8	67.92	74.78	38.5 ^abc^	45.53 ^ab^	37.00
9	66.73	74.38	36.55 ^ab^	56.20 ^bc^	38.24
10	72.50	76.51	56.60 ^d^	47.49 ^ab^	27.22
11	67.36	75.90	33.62 ^ab^	45.96 ^ab^	29.58
12	73.74	80.44	53.42 ^cd^	50.86 ^bc^	37.13
13	66.75	74.65	37.86 ^ab^	45.65 ^ab^	28.26
14	65.10	71.39	29.58 ^a^	34.63 ^a^	19.73
SEM	2.68	2.15	4.75	4.86	5.64
*p*-value	0.718	0.607	0.045	0.042	0.659

^a,b,c,d^ Means within columns not sharing a common suffix are significantly different at the 5% level of probability. Here, treatment 14 was not a part of the response surface analysis; it was only added to compare the results with other treatments.

## Data Availability

The research data supporting this study will be shared upon a reasonable request made to the corresponding author. The data are not publicly available due to privacy.

## References

[1] Zhang N., Wang M., Wang N. (2002). Precision agriculture—A worldwide overview. Comput. Electron. Agric..

[2] Moss A., Parkinson G., Crowley T., Pesti G. (2021). Alternatives to formulate laying hen diets beyond the traditional least-cost model. J. Appl. Poult. Res..

[3] Wilkinson S. Big data for poultry–what is possible. Proceedings of the 29th Annual Australian Poultry Science Symposium.

[4] Molnár A., Hamelin C., Delezie E., Nys Y. (2018). Sequential and choice feeding in laying hens: Adapting nutrient supply to requirements during the egg formation cycle. World’s Poult. Sci. J..

[5] Moss A.F., Dao T.H., Crowley T.M., Wilkinson S.J. (2023). Interactions of diet and circadian rhythm to achieve precision nutrition of poultry. Anim. Prod. Sci..

[6] Moran E.T. (1987). Protein requirement, egg formation and the hen’s ovulatory cycle. J. Nutr..

[7] Silver R. (1986). Circadian and interval timing mechanisms in the ovulatory cycle of the hen. Poult. Sci..

[8] Penz J.A., Jensen L. (1991). Influence of protein concentration, amino acid supplementation, and daily time of access to high-or low-protein diets on egg weight and components in laying hens. Poult. Sci..

[9] Hiramoto K., Muramatsu T., Okumura J. (1990). Protein synthesis in tissues and in the whole body of laying hens during egg formation. Poult. Sci..

[10] Dijkstra J., Kebreab E., Kwakkel R., France J. (2006). Development of a dynamic model of calcium and phosphorus flows in layers. Nutrient Digestion and Utilization in Farm Animals: Modelling Approaches.

[11] Kebreab E., France J., Kwakkel R., Leeson S., Kuhi H.D., Dijkstra J. (2009). Development and evaluation of a dynamic model of calcium and phosphorus flows in layers. Poult. Sci..

[12] De Los Mozos J., Gutierrez del Alamo A., Gerwe T.v., Sacranie A., Perez de Ayala P. Oviposition feeding compared to normal feeding: Effect on performance and egg shell quality. Proceedings of the 23rd Annual Australian Poultry Science Symposium.

[13] Leeson S., Summers J. (2009). Commercial Poultry Nutrition.

[14] Bryden W.L., Li X., Ruhnke I., Zhang D., Shini S. (2021). Nutrition, feeding and laying hen welfare. Anim. Prod. Sci..

[15] Li X., Zhang D., Yang T.Y., Bryden W.L. (2016). Phosphorus bioavailability: A key aspect for conserving this critical animal feed resource with reference to broiler nutrition. Agriculture.

[16] Waldroup P.W., Hellwig H. (2000). The potential value of morning and afternoon feeds for laying hens. J. Appl. Poult. Res..

[17] Molnár A., Kempen I., Sleeckx N., Zoons J., Maertens L., Ampe B., Buyse J., Delezie E. (2018). Effects of split feeding on performance, egg quality, and bone strength in brown laying hens in aviary system. J. Appl. Poult. Res..

[18] El-kelawy M. (2020). Effect of split feeding system on egg production and egg quality of dandarawi layers. Egypt. Poult. Sci. J..

[19] Traineau M., Bouvarel I., Mulsant C., Roffidal L., Launay C., Lescoat P. (2015). Modulation of energy and protein supplies in sequential feeding in laying hens. Animal.

[20] van Emous R., Mens A. (2021). Effects of twice a day feeding and split feeding during lay on broiler breeder production performance, eggshell quality, incubation traits, and behavior. Poult. Sci..

[21] Svihus B., Itani K. (2019). Intestinal passage and its relation to digestive processes. J. Appl. Poult. Res..

[22] Desbruslais A., Wealleans A., Gonzalez-Sanchez D., di Benedetto M. (2021). Dietary fibre in laying hens: A review of effects on performance, gut health and feather pecking. World’s Poult. Sci. J..

[23] Hartini S., Choct M., Hinch G., Kocher A., Nolan J. (2002). Effects of light intensity during rearing and beak trimming and dietary fiber sources on mortality, egg production, and performance of ISA brown laying hens. J. Appl. Poult. Res..

[24] Van Krimpen M., Kwakkel R., Van der Peet-Schwering C., Den Hartog L., Verstegen M. (2009). Effects of nutrient dilution and nonstarch polysaccharide concentration in rearing and laying diets on eating behavior and feather damage of rearing and laying hens. Poult. Sci..

[25] Faruk M.U., Bouvarel I., Même N., Rideau N., Roffidal L., Tukur H.M., Bastianelli D., Nys Y., Lescoat P. (2010). Sequential feeding using whole wheat and a separate protein-mineral concentrate improved feed efficiency in laying hens. Poult. Sci..

[26] Faruk M.U., Bouvarel I., Même N., Roffidal L., Tukur H., Nys Y., Lescoat P. (2010). Adaptation of wheat and protein-mineral concentrate intakes by individual hens fed ad libitum in sequential or in loose-mix systems. Br. Poult. Sci..

[27] Keshavarz K. (1998). Further investigations on the effect of dietary manipulation of protein, phosphorus, and calcium for reducing their daily requirement for laying hens. Poult. Sci..

[28] Keshavarz K. (1998). Investigation on the possibility of reducing protein, phosphorus, and calcium requirements of laying hens by manipulation of time of access to these nutrients. Poult. Sci..

[29] Lee K., Ohh Y. (2002). Effects of nutrient levels and feeding regimen of am and pm diets on laying hen performances and feed cost. Korean J. Poult. Sci..

[30] Mozos J.d.l., Gutierrez del Alamo A., Gerwe T.V., Sacranie A., Perez de Ayala P. Oviposition feeding: Effect of reduced energy and protein levels on performance of laying hens. Proceedings of the 23rd Annual Australian Poultry Science Symposium.

[31] Mens A., Van Krimpen M., Kwakkel R. (2020). Nutritional approaches to reduce or prevent feather pecking in laying hens: Any potential to intervene during rearing?. World’s Poult. Sci. J..

[32] Nasr M., Murrell J., Nicol C. (2013). The effect of keel fractures on egg production, feed and water consumption in individual laying hens. Br. Poult. Sci..

[33] De Leon A., Kidd M., Corzo A. (2010). Box-Behnken Design: Alternative multivariate design in broiler nutrition research. World’s Poult. Sci. J..

[34] NHMRC (2013). Australian Code of Practice for the Care and Use of Animals for Scientific Purposes.

[35] Liu S.Y., Naranjo V.D., Chrystal P.V., Buyse J., Selle P.H. (2019). Box-Behnken optimisation of growth performance, plasma metabolites and carcass traits as influenced by dietary energy, amino acid and starch to lipid ratios in broiler chickens. PLoS ONE.

[36] Liu S.Y., Sydenham C., Selle P. (2016). Feed access to, and inclusions of fishmeal and corn starch in, sorghum-based broiler diets influence growth performance and nutrient utilisation as assessed by the Box-Behnken response surface design. Anim. Feed Sci. Technol..

[37] AOAC—Association of Official Analytical Chemists (1990). Official Methods of Analysis of AOAC International.

[38] Dumas J. (1831). Procédés de l’analyse organique. Ann. Chim. Phys..

[39] Zanu H., Kheravii S., Morgan N., Bedford M., Swick R. (2020). Interactive effect of dietary calcium and phytase on broilers challenged with subclinical necrotic enteritis: 3. Serum calcium and phosphorus, and bone mineralization. Poult. Sci..

[40] Kong C., Adeola O. (2014). Evaluation of amino acid and energy utilization in feedstuff for swine and poultry diets. Asian-Australas. J. Anim. Sci..

[41] Castro F., Chai L., Arango J., Owens C., Smith P., Reichelt S., DuBois C., Menconi A. (2023). Poultry industry paradigms: Connecting the dots. J. Appl. Poult. Res..

[42] Kidd M., Tillman P., Tillman N., Chrystal P. (2023). Precision feeding for optimizing poultry production. CABI Rev.

[43] Pomar C., Van Milgen J., Remus A. (2019). 18: Precision livestock feeding, principle and practice. Poultry and Pig Nutrition: Challenges of the 21st Century.

[44] Spring P. (2013). The challenge of cost effective poultry and animal nutrition: Optimizing existing and applying novel concepts. Lohmann Inf..

[45] Thirumalaisamy G., Muralidharan J., Senthilkumar S., Hema Sayee R., Priyadharsini M. (2016). Cost-effective feeding of poultry. Int. J. Sci. Environ. Technol..

[46] Pousga S., Boly H., Ogle B. (2005). Choice feeding of poultry: A review. Livest. Res. Rural Dev..

[47] De los Mozos J., Sacranie A., Van Gerwe T. Performance and eggshell quality in laying hens fed two diets through the day with different levels of calcium or phosphorous. Proceedings of the 25th Annual Australian Poultry Science Symposium.

[48] Hy-line-Brown-International (2014). Hy-Line Brown Conventional Systems Performance Guide. https://www.hyline.com/filesimages/Hy-Line-Products/Hy-Line-Product-PDFs/Brown/BRN%20STD%20ENG.pdf.

[49] Shini A., Shini S., Bryden W. (2019). Fatty liver haemorrhagic syndrome occurrence in laying hens: Impact of production system. Avian Pathol..

[50] Trott K., Giannitti F., Rimoldi G., Hill A., Woods L., Barr B., Anderson M., Mete A. (2014). Fatty liver hemorrhagic syndrome in the backyard chicken: A retrospective histopathologic case series. Vet. Pathol..

[51] Saki A., Rahmani A., Yousefi A. (2019). Calcium particle size and feeding time influence egg shell quality in laying hens. Acta Scientiarum. Anim. Sci..

[52] An S., Kim D., An B.K. (2016). Effects of dietary calcium levels on productive performance, eggshell quality and overall calcium status in aged laying hens. Asian-Australas. J. Anim. Sci..

[53] Londero A., Rosa A., Giacomini C., Vivas C., Orso C., De Freitas H., Gressler L., Vargas A. (2015). Effect of different feeding schedules on reproductive parameters and egg quality of broiler breeders. Anim. Feed Sci. Technol..

[54] Van Emous R. (2023). Effects of feeding strategies during lay on broiler breeder production performance, eggshell quality, incubation traits, and behavior. Poult. Sci..

[55] Lagos L.V., Lee S.A., Fondevila G., Walk C.L., Murphy M.R., Loor J.J., Stein H.H. (2019). Influence of the concentration of dietary digestible calcium on growth performance, bone mineralization, plasma calcium, and abundance of genes involved in intestinal absorption of calcium in pigs from 11 to 22 kg fed diets with different concentrations of digestible phosphorus. J. Anim. Sci. Biotechnol..

